# A sandwich-like configuration with a signal amplification strategy using a methylene blue/aptamer complex on a heterojunction 2D MoSe_2_/2D WSe_2_ electrode: Toward a portable and sensitive electrochemical alpha-fetoprotein immunoassay

**DOI:** 10.3389/fcimb.2022.916357

**Published:** 2022-10-27

**Authors:** Supakeit Chanarsa, Jaroon Jakmunee, Kontad Ounnunkad

**Affiliations:** ^1^ Department of Chemistry, Faculty of Science, Chiang Mai University, Chiang Mai, Thailand; ^2^ Center of Excellence for Innovation in Chemistry, Faculty of Science, Chiang Mai University, Chiang Mai, Thailand; ^3^ Research Center on Chemistry for Development of Health Promoting Products from Northern Resources, Chiang Mai University, Chiang Mai, Thailand

**Keywords:** alpha-fetoprotein, liver cancer, methylene blue/aptamer complex, sandwich-like immunosensor, electrochemical sensor, 2D MoSe_2_/2D WSe_2_, portable immunoassay, portable immunosensor

## Abstract

Liver cancer is one of the most common global health problems that features a high mortality rate. Alpha-fetoprotein (AFP) is a potential liver cancer biomarker for the diagnosis of liver cancer. The quantitative detection of AFP at an ultratrace level has important medical significance. Using the reaction of the antibody–antigen pair in an immunosensor enables the sensitive and selective AFP assay. Finding a strategy in signal generation and amplification is challenging to fabricate new sensitive electrochemical immunosensors for AFP detection. This study demonstrates the construction of a simple, reliable, and label-free immunosensor for the detection of AFP on a smart phone. Exfoliated two-dimensional (2D) molybdenum diselenide (MoSe_2_) and 2D tungsten diselenide (WSe_2_) were employed to modify the disposable screen-printed carbon electrode (SPCE) to use as the electrochemical platform, which is affixed to a small potentiostat connected to a smart phone. The modified electrode offers antibody immobilization and allows detection of AFP *via* an immunocomplex forming a sandwich-like configuration with the AFP-corresponding aptamer. A heterojunction 2D MoSe_2_/2D WSe_2_ composite improves the SPCE’s reactivity and provides a large surface area and good adsorption capacity for the immobilizing antibodies. The signal generation for the immunosensor is from the electrochemical response of methylene blue (MB) intercalating into the aptamer bound on the electrode. The response for the proposed sandwich-like immunosensor is proportional to the AFP concentration (1.0–50,000 pg ml^-1^). The biosensor has potential for the development of a simple and robust point-of-care diagnostic platform for the clinical diagnosis of liver cancer, achieving a low limit of detection (0.85 pg ml^-1^), high sensitivity, high selectivity, good stability, and excellent reproducibility.

## Introduction

Alpha-fetoprotein (AFP) is a tumor marker that is often used in the diagnosis and treatment of primary liver cancer (Zhang et al., 2007). AFP is an oncofetal glycoprotein with a molecular weight of 70,000 Da (Jiang et al., 2016). The AFP level in healthy human serum is less than 25 ng ml^-1^, but it rises dramatically in individuals with liver cancer (Jiang et al., 2010; Liu et al., 2011; Su et al., 2011). In general, enzyme-linked immunosorbent assay (ELISA) (Darwish et al., 2013), radioimmunoassay (Shafik et al., 2014), chemiluminescence immunoassay (Fu et al., 2006), and electrochemical immunoassay (Yuan et al, 2017) can be employed to detect such biomarkers. The first three standard immunoassays, on the other hand, have several disadvantages. They require expensive instrumentation and skilled operators (Wang et al., 2014). Moreover, they need costly specialized consumables, including sample well plates, chips, and reagent kits, and hence this has limited their use for clinical point-of-care (POC) applications in circumstances with minimal resources (Cristea et al., 2015). Electrochemical immunosensors have overcome these drawbacks, the measurements of which are based on specific antigen–antibody interactions (Wang et al., 2017; Putnin et al., 2018; Pei et al., 2019; Upan et al., 2020; Zhao et al., 2020). In addition, they can be operated with simplicity, high selectivity, high sensitivity, and good stability (Pei et al., 2019; Zhao et al., 2019). During the detection of target analytes, the immunocomplex generally forms on the sensing surface *via* molecular recognition by primary antibody and then signal amplification is performed by the formation of a sandwich immunocomplex with another detection or secondary antibody tagged with enzymes (Zhong et al., 2015; Shen et al., 2020), metals (Wei et al., 2016; Liu et al., 2017; Wang et al., 2018), nanoparticle nanotags (Wei et al., 2016; Putnin et al., 2019; Zhao et al., 2020; Xiao et al., 2021), and redox probes (Gao et al., 2014; Rong et al., 2021). The preparation of labeled antibodies requires many steps and costly chemicals; moreover, tagging with biomolecules would cause instability in the detection because they are environmentally sensitive. Instead of the use of labeling particles, the redox probe/aptamer complex is exploited for signal amplification (Taleat et al., 2014) because the aptamer has high specificity for and high affinity with the target analyte (Huang et al., 2008; Wang et al., 2008). In recent years, aptamers, small synthetic single-strand deoxyribonucleic acid (DNA) or ribonucleic acid (RNA) molecules, have been found to be recognition elements on the sensing surface (Han et al., 2010; Mazloum et al., 2019), which can be developed as an aptasensor (Han et al., 2010; Mazloum et al., 2019; Upan et al., 2021). They offer superior thermal and chemical stability, great repeatability, and outstanding stability (Wang et al., 2008; Farzadfard et al., 2020). Furthermore, they have shown ease of manufacture, good controllability, facile large-scale synthesis, and adaptability (Taleat et al., 2014). For signal amplification using an aptamer, it forms a sandwich-like structure after binding to the target analyte and then the redox species can selectively intercalate into its DNA or RNA structure, thus giving a current response that can be monitored regarding the target concentration (Taleat et al., 2014). The electrochemical indicator, namely, methylene blue (MB), has been utilized to investigate protein–aptamer interactions. At an electrode surface, MB is electrochemically converted to leucomethylene blue (LB) by absorbing two electrons. As a result, this indication has been employed to identify protein–aptamer interactions (Wang et al., 2010; Yan et al., 2011). In addition, a DNA aptamer contains a lot of G bases that can absorb MB. MB can particularly attach to G bases of the ss-DNA aptamer. To circumvent the use of costly labeling, MB reacts directly with the aptamer or DNA (Li et al., 2007; Lin et al., 2007), forming an electrochemically detectable MB/aptamer complex.

Two-dimensional (2D) transition metal dichalcogenides (TMDs) play many important roles in many fields involving photovoltaics (Wang et al., 2018; Das et al., 2019), sensors (Rohaizad et al., 2017; Jiang et al., 2020; Yaiwong et al., 2021; Pothipor et al., 2022), catalysts (Harvey et al., 2015; Sakthivel et al., 2018), and energy storage devices (Bissett et al., 2016; Liu et al., 2016). They can be simply exfoliated by using organic solvents (Backes et al., 2017; Synnatschke et al., 2019). They possess atomically layered structures, large surface areas, and outstanding electrochemical characteristics (Jing et al., 2014; Rohaizad et al., 2017). In their uses in electrochemical sensors, 2D materials can not only improve electrode reactivity but also load chemicals and biochemicals at high contents because of unique electrochemical properties and massive electroactive surface areas (Yaiwong et al., 2021; Pothipor et al., 2022). They are getting a lot of interest as both electrode modifiers (Rohaizad et al., 2017; Yaiwong et al., 2021) and tagging materials (Hong et al., 2020) for immunosensors. They also present fast heterogeneous electron-transfer rates (Rohaizad et al., 2017). Recently, electrochemical detection utilizing a 2D MoS_2_ and graphene oxide (GO)-modified electrode has been carried out with good sensitivity, high selectivity, and high stability (Yaiwong et al., 2021). It was found that a single 2D WX_2_ offers better analytical performance than that of a single 2D MoX_2_ when they are incorporated into electrochemical glucose biosensors (Rohaizad et al., 2017). Nevertheless, the sensing applications employing the combination of each material or their heterojunctions are still in the infancy stage. Since some reports show a synergistic effect, which is observed with GO/2D material mixtures, many interests focus on the construction of sensors from such materials (Yaiwong et al., 2021; Pothipor et al., 2022). There is no report about the use of 2D inorganic compound heterojunction such as from 2D molybdenum diselenide (MoSe_2_) and 2D tungsten diselenide (WSe_2_) in developing an electrochemical sensor, especially a biosensor. The two components are expected to synergistically contribute to good device performance. To demonstrate the full functionality of the electrochemical immunosensing system, miniaturization and convenient integration into small-size sensors are needed for the practical POC fields (Wang et al., 2015; Zhang et al., 2016). The design of the portable and lightweight electrochemical detection system is challenging. Furthermore, a printed electrode often allows the construction of portable and disposable sensor devices (Upan et al., 2020; Phetsang et al., 2021; Pothipor et al, 2022). For example, a small electrochemical cell can be made by printing three different printed electrodes on a single platform. Generally, the cell is inserted into a small portable potentiostat or affixed to a smart phone (Ainla et al., 2018; Anshori et al., 2022). The printed electrode such as a screen-printed electrode (SPE) can be widely functionalized and modified with various kinds of materials, electrode modifiers, and sensing elements such as active molecules, enzymes, antibodies, recepters, and aptamers. It has the simplicity of use for fabrication of a variety of electrochemical sensors and biosensors.

In this work, a portable immunosensor with the sandwich-like configuration has been constructed for the detection of AFP for the first time. AFP is chosen as the model target analyte. Instead of the use of labeling particles, the strategy employs an MB/aptamer complex for signal generation and amplification. The sensor performs the sandwich-like immunoassay on a carbon working area of the commercial three-electrode system SPE, modified with 2D MoSe_2_/2D WSe_2_ heterojunction. The combination of each component offers high analytical performances in detecting AFP in human serum. The immunoassay is carried out on a small potentiostat attached to a smart phone (Android). A 2D MoSe_2_/2D WSe_2_ composite improves the SPE’s electrochemical reactivity and is a kind of excellent 2D nanomaterial with a high surface capacity and favorable biocompatibility. The captured anti-AFP antibody is sufficiently immobilized on the 2D MoSe_2_/2D WSe_2_ surface. After the AFP target protein is bound on the immunosensing surface, the subsequent additional intercalation of MB is caused by the aptamer coverage on the immunocomplex. With the presence of the aptamer, the MB uptake on the immunosensor is extremely increased as seen in its higher oxidation peak current response observed by differential pulse voltammetric measurements. As a result, the electrochemical response is linearly proportional to a wide range of AFP concentrations and offers good detection ability with a low limit of detection (LOD). Other analytical parameters such as specificity, reproducibility, and stability are studied. This developed immunosensor could be applied to screening and monitoring of AFP associated with liver cancer.

## Experiment

### Chemicals and materials

MoSe_2_ (325 mesh, 99.9%) and N-methyl-2-pyrrolidone (≥99.0%) were purchased from Sigma-Aldrich (USA). WSe_2_ (99.8%) was purchased from Alfa Aesar (Lancashire, UK). Phosphate-buffered saline (PBS) tablets (pH 7.4), dopamine hydrochloride (DA; 99.5%), immunoglobulin G (IgG) from human serum (4.8 mg ml^-1^, ≥95%), interleukin-6 (IL-6; lot: 0409AFC16, ≥98%), and myoglobin (Mb) from human heart (≥95%) were bought from Sigma-Aldrich (Singapore). Anti-human AFP antibody and AFP were purchased from Fitzgerald (USA). Potassium ferrocyanide {K_4_[Fe(CN)_6_]} was ordered from Scharlau (Spain), while potassium ferricyanide {K_3_[Fe(CN)_6_]} was purchased from Merck (Germany). L(+)-ascorbic acid (AA; 99.7%) was bought from Merck (Germany). Glucose (Glu; 99%) was bought from Fluka (Switzerland). IL-15 (lot: 2381730, ≥98%) was bought from Millipore (Burlington, MA, USA). Uric acid (UA; 98.5%) and MB (>97.0) were purchased from Sigma-Aldrich (St. Louis, MO, USA). Mucin1 protein (MUC1; lot: LC08JA2304) was purchased from Sino Biological Inc. (China). Aptamer 5′-GTGACGCTCCTAACGCTGACTCAGG-TGCAGTTCTCGACTCGGTCTT-3′ was bought from Sigma-Aldrich (Singapore).

### Apparatus and Methods

**Scheme 1:**
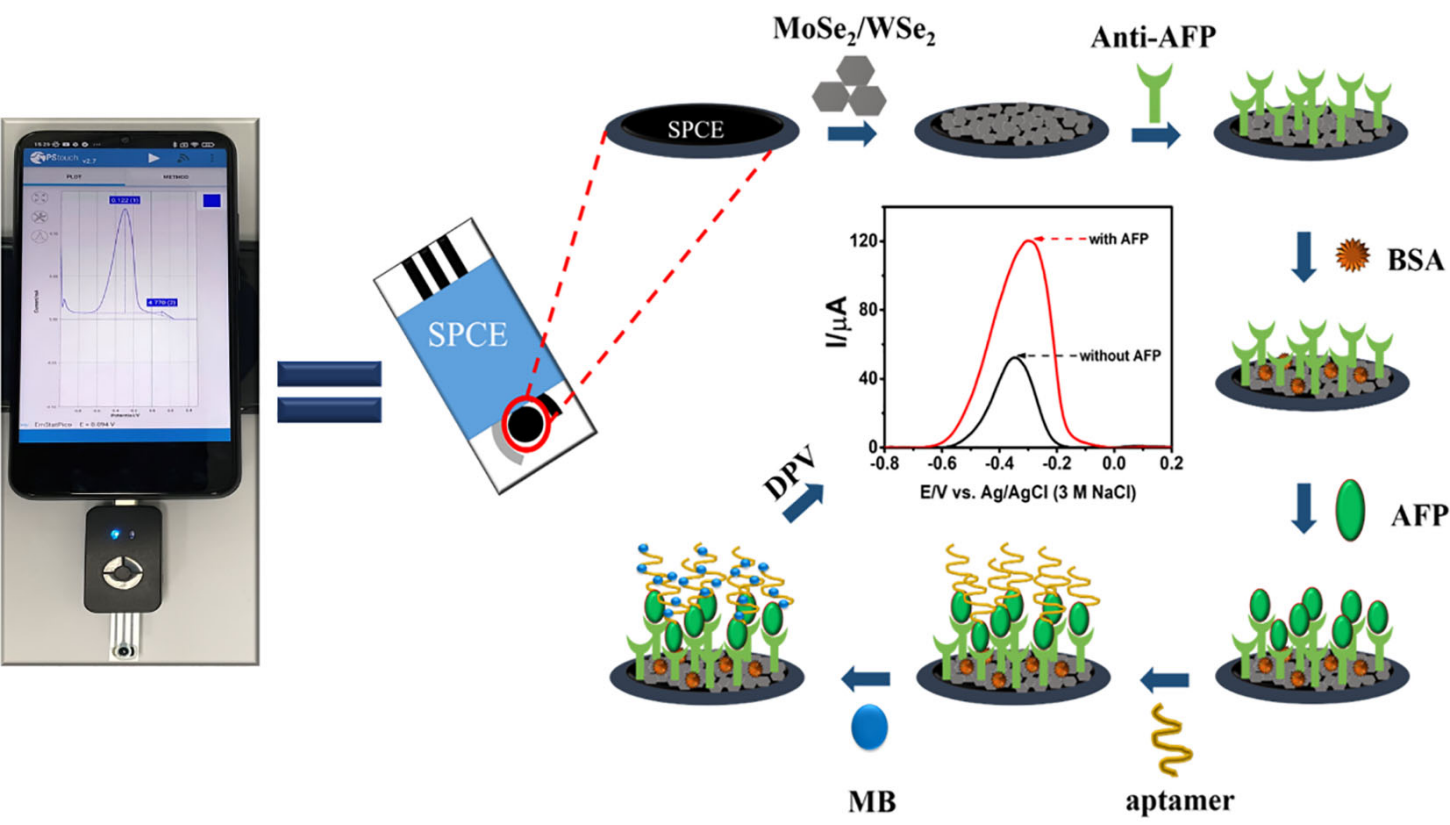
Fabrication and AFP detection process of the electrochemical immunosensor.

The electrochemical measurements involving differential pulse voltammetry (DPV), cyclic voltammetry (CV), and electrochemical impedance spectroscopy (EIS) were performed using EmStat3, Sensit/Smart, and PalmSens4 potentiostats (PalmSens, Netherlands). For the measurements in a 5-ml electrochemical cell, a platinum (Pt) wire counter electrode, a silver/silver chloride (Ag/AgCl, 3M NaCl) reference electrode, and a homemade working screen-printed carbon electrode (SPCE) were used. The homemade SPCEs as electrode support were employed for material characterization and optimization of sensor fabrication parameter. Pt and Ag/AgCl electrodes were obtained from Nilaco Co. Ltd. (Tokyo, Japan) and Bioanalytical Systems, Inc. (IN, USA), respectively. For the construction of the new proposed sensor, ItalSens three-electrode SPEs were employed and purchased from PalmSens, Netherlands. Micrographs of the surface morphologies for the modified electrodes were recorded on a JEOL scanning electron microscope (SEM; JSM-IT300, Tokyo, Japan) equipped with a JEOL energy-dispersive X-ray spectrometer (EDX; JSM-IT300LV, Japan). The morphologies of 2D materials were investigated using a JEOL transmission electron microscope (TEM; JEM-2010, Japan). For measuring the DPV current response of the immunosensor, an ItalSens SPE affixed to the Sensit/Smart small potentiostat device, on which a drop of electrolyte was coated, was operated on a Redmi (Xiaomi) smart phone **Scheme 1**.

### Preparation of 2D MoSe_2_ and 2D WSe_2_ powders

Bulk MoSe_2_ and WSe_2_ powders (1.0 g) were separately crushed in a mortar for 20 min. Then, 600 ml of N-methyl-2- pyrrolidine was poured into the powder, and the mixture was ground for 40 min. The resultant mixture was stored at an ambient temperature for 48 h. The MoSe_2_ and WSe_2_ solutions were then sonicated for 1.5 h before being dried in an oven at 60°C overnight to achieve 2D MoSe_2_ and 2D WSe_2_ powders.

### Fabrication of the sandwich-like immunosensor on a MoSe_2_/WSe_2_-modified SPE

To begin with the dispersion of 2D nanomaterials, 3.0 mg mixture of 2D MoSe_2_ (75%) and 2D WSe_2_ (25%) powders in 1.0 ml of deionized water was bath-sonicated for 90 min. Before modification with the 2D materials, the working SPCE of the commercial SPE was treated in a plasma chamber (Putnin et al., 2018; Upan et al., 2020; Upan et al., 2021; Yaiwong et al., 2021; Pothipor et al., 2022). Then, 2.5 µl of 2D MoSe_2_/2D WSe_2_ composite dispersion was dropped onto a plasma-cleaned SPCE, and the modified SPE was dried at room temperature. In the following modifications, each incubation was done in a humidity chamber at room temperature. The modified electrode was incubated with 5.0 µl of anti-AFP (50 µg ml^-1^) in 0.010 M PBS buffer for 40 min, washed with the PBS buffer several times to remove free antibody, and dried at room temperature. To generate a nonspecific adsorption-free electrode surface, the anti-AFP/MoSe_2_/WSe_2_-modified SPE was incubated with 5.0 µl of 1.0% w/w bovine serum albumin (BSA) for 40 min and washed with PBS several times. After that, the prepared electrodes were incubated with 5.0 µl of AFP solutions at different concentrations (1–50,000 pg ml^-1^) for 40 min and again washed with PBS several times. The electrode surface was rinsed with 0.010 M PBS buffer many times after incubation with 2.0 µl of 10 µM aptamer solution for 40 min. To measure the analytical current responses, 5.0 µl of 10 mM MB solution was dropped onto the resultant SPE for 30 min. Excess MB was removed by repeatedly washing the electrode with 0.010 M PBS buffer three times. The electrochemical signals in PBS buffer (pH 7.4) were recorded using DPV with a step potential of 10 mV, a modulation amplitude of 50 mV, a modulation period of 10 ms, a scan rate of 50 mV s^-1^, and potential scan ranges from -0.50 to 0.10 V (for homemade SPCE) and from -0.80 to 0.20 V (for commercial SPE) at room temperature. The fabrication steps are shown in **Scheme 1**. For all electrochemical experiments, data for each condition were obtained with five replicates.

## Results and discussion

### Morphological characteristics

**Figure 1:**
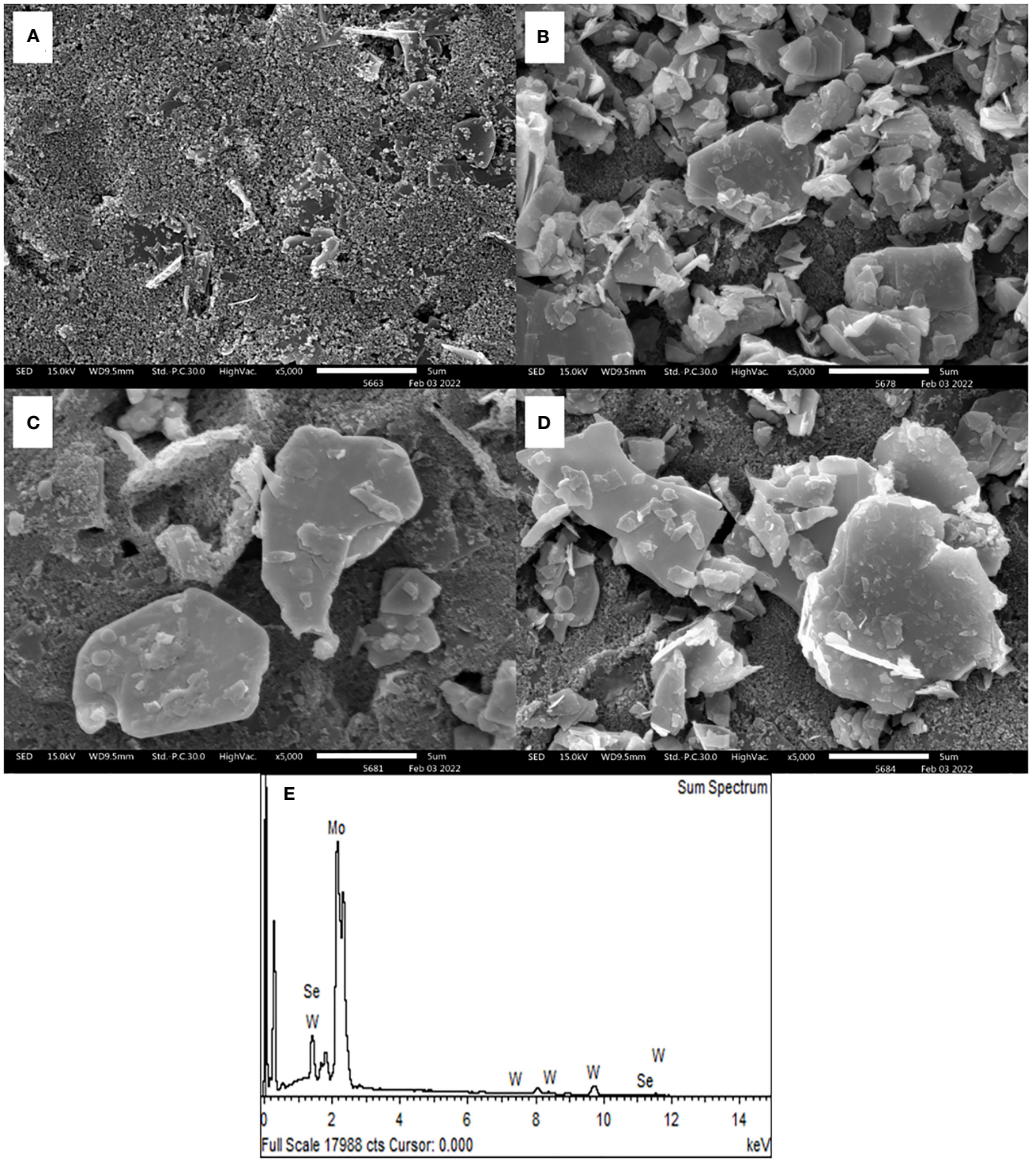
SEM photographs of **(A)** naked SPCE and SPCEs modified with 2D MoSe_2_ **(B)**, 2D WSe_2_ **(C)**, and 2D MoSe_2_/2D WSe_2_ **(D)**. **(E)** EDX spectrum of 2D MoSe_2_/2D WSe_2_ composite-modified SPCE.

SEM observation was used to evaluate the morphologies of bare SPCE and 2D MoSe_2_-, 2D WSe_2_-, and 2D MoSe_2_/2D WSe_2_-modified SPCEs. **Figure 1** shows an SEM image of SPCE. It contains the small and large conductive graphite particles; the small particles cover the big particles. After the SPCE was modified with 2D MoSe_2_ and 2D WSe_2_, the surface was partly coated with platelet-like particles as illustrated in **Figures 1B** and **C**, respectively. The larger particles were observed with the 2D WSe_2_-modified SPCE. The existence of both 2D MoSe_2_ and 2D WSe_2_ crystallites with straight-cut edges and disordered arrangement is on the 2D MoSe_2_/2D WSe_2_-modified SPCE as depicted in **Figure 1** (Sajedi-Moghaddam et al., 2019). **Figure 1** depicts the EDX spectrum of 2D MoSe_2_/2D WSe_2_-modified SPCE’s surface, indicating the presence of Mo, W, and Se elements. The sheet structures of the 2D materials are confirmed using TEM images as presented in **Figure S1**.

### Electrochemical characterization

**Figure 2:**
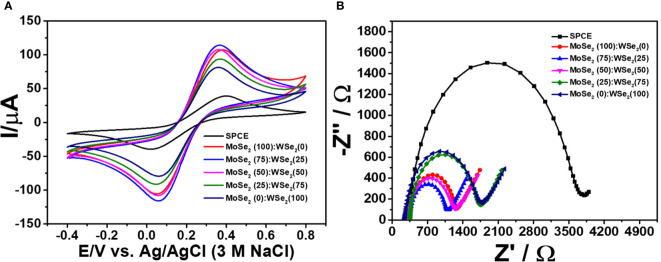
**(A)** Cyclic voltammograms and **(B)** EIS spectra of bare and modified SPCEs in contact with 0.010 M PBS containing 5.0 mM K_3_[Fe(CN)_6_]/K_4_[Fe(CN)_6_].

In the development of the immunosensor using novel nanomaterials, electrode kinetics is crucial. The electrode with fast kinetics is especially attractive for use as an electrochemical transducer. Its corresponding sensors offer good detection efficiency. In general, the electrode is improved by coating with electroactive compounds of interest in order to achieve such property. In this study, the bare SPCE and SPCEs covered with single and bicomponent 2D materials, namely, 2D MoSe_2_, 2D WSe_2_, and 2D MoSe_2_/2D WSe_2_ composites with the ratios of 75:25, 50:50, and 25:75, were then electrochemically characterized using CV and EIS in 0.010 M PBS containing 5.0 mM K_3_[Fe(CN)_6_]/K_4_[Fe(CN)_6_]. The rate of heterogeneous electron transfer (*k*_0_) is inversely related to the peak-to-peak separation (Δ*E*_p_) and charge-transfer resistance (*R*_ct_) of the electrochemical reaction at the working electrode. Cyclic voltammograms obtained for 2D MoSe_2_-, 2D WSe_2_-, and 2D MoSe_2_/2D WSe_2_ composite-modified SPCEs are shown in **Figure 2**. The improved current response of [Fe(CN)_6_]^3-/4-^ reaction is found at modified SPCEs. The lower Δ*E*_p_ values observed are 0.31, 0.30, 0.29, 0.30, and 0.30 V for SPCEs modified with 2D MoSe_2_, 2D WSe_2_, and 2D MoSe_2_/2D WSe_2_ samples with the ratios of 75:25, 50:50, and 25:75, respectively, as compared to that of naked SPCE (0.38 V). This suggests that modification with all 2D materials can mediate the electron transfer (Tan et al., 2016; Rojas et al., 2020). Although the Δ*E*_p_ values for the modified SPCEs are insignificantly different, the SPCE based on 2D MoSe_2_/2D WSe_2_ with the ratio of 75:25 shows the best current response (ca. 117 μA, 3-fold improvement). Additionally, an experiment using an EIS was also conducted to determine the resistances of the modified electrodes. The EIS spectra are presented as Nyquist plots. A spectrum represents semicircular and linear components, which are related to the kinetic and mass transfer controls at high and low frequencies, respectively (Petsawi et al., 2019; Chanarsa et al., 2020; Pothipor et al., 2022). Small semicircles mean low *R*_ct_. **Figure 2** shows Nyquist plots of bare SPCE and MoSe_2_-, WSe_2_-, and MoSe_2_/WSe_2_-modified SPCEs in contact with 5.0 mM K_3_[Fe(CN)_6_]/K_4_[Fe(CN)_6_] in 0.010 M PBS. It is found that after coating with the 2D materials and their mixtures on SPCE, the semicircle decreases, indicating a faster electron-transfer process. The *R*_ct_ value of the bare SPCE is 3,266 Ω, while *R*_ct_ values of the electrodes modified with 2D MoSe_2_, 2D WSe_2_, and 2D MoSe_2_/2D WSe_2_ (75:25, 50:50, 25:75) composites are 906, 1,397, 727, 868, and 1,392 Ω, respectively. All modified electrodes reveal lower resistances as compared to that of bare SPCE (Cunningham et al., 2015). This agrees well with the CV result. As seen in this figure, 2D WSe_2_ offers lower resistance than that of bare SPCE, but it presents higher resistance than that of 2D MoSe_2_. Interestingly, at the optimal composition (75:25 for 2D MoSe_2_:2D WSe_2_), both materials synergistically work with the best electron-transfer process, implying the best electrode kinetics. This composition also gives the highest current response in the CV measurement. It is plausible that the 2D MoSe_2_/2D WSe_2_ (75:25) composite on the modified SPCE would produce a favorable surface with good electrical conductivity/electrochemical reactivity and a large surface area. Among all of the modified electrodes, it displays the lowest *R*_ct_ value that would in turn govern the charge mobility on the sensing surface, thus resulting in high sensitivity in sensors. Therefore, for fabrication of the sandwich-like immunosensor, the 2D MoSe_2_/2D WSe_2_ (75:25)-modified SPCE is a good candidate.

### Study of the fabrication steps

**Figure 3:**
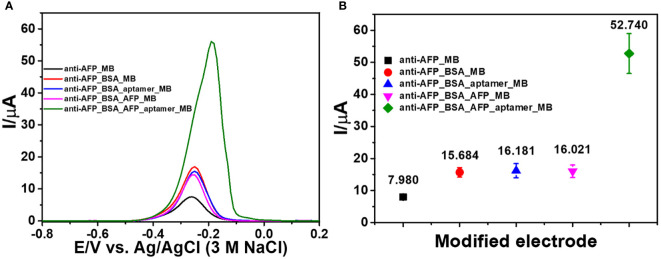
**(A)** DPV responses of the modified electrodes in contact with 0.010 M PBS (pH 7.4) after each fabrication step of the sandwich-like immunosensor and **(B)** corresponding peak currents of the modified electrodes.

The related differential pulse voltammogram (DPV) was recorded using MB as a redox probe in order to monitor each preparation stage of the immunosensor. **Figure 3** depicts the DPVs of electrodes at each construction step, and the corresponding currents are shown in **Figure 3**. Considering the redox response of MB at a potential of 0.27 V, all electrodes have different adsorption abilities for MB. The small oxidation peak current (black line) is caused by the 2D MoSe_2_/2D WSe_2_-modified SPCE’s adsorption ability of electroactive MB after it was incubated with the anti-AFP solution. The red line shows the MB current response of the anti-AFP/2D MoSe_2_/2D WSe_2_-modified SPCE electrode after coating with BSA and then MB. The peak current increases because of its higher adsorption capacity for MB. The blue line shows that an insignificant current change is observed when the anti-AFP/MoSe_2_/WSe_2_-modified SPCE was sequentially incubated with solutions without AFP, and aptamer and MB, indicating the absence of AFP and aptamer on the electrode surface. Without AFP, the aptamer could not bind to the electrode surface and its nonspecific adsorption could not occur. This suggests the character of the anti-AFP antibody. After AFP (50 ng ml^-1^) was immobilized on the sensing electrode surface without the addition of aptamer, no significant change in the peak current of MB (pink line) is found. Interestingly, the anti-AFP/2D MoSe_2_/2D WSe_2_-modified SPCE electrode incubated with solutions of BSA, AFP, aptamer, and MB, respectively, significantly improved the current (green line), resulting from MB’s selective intercalation in the aptamer structure. This indicates high affinity of the aptamer for MB. According to the high current response of MB caused by the occurrence of the aptamer’s binding to the captured target AFP, the current intensity would be related to the amount of target AFP on the sensing surface. Therefore, the proposed sandwich-like immunosensor can be used to detect AFP at trace levels.

### Optimization of fabrication conditions

**Figure 4:**
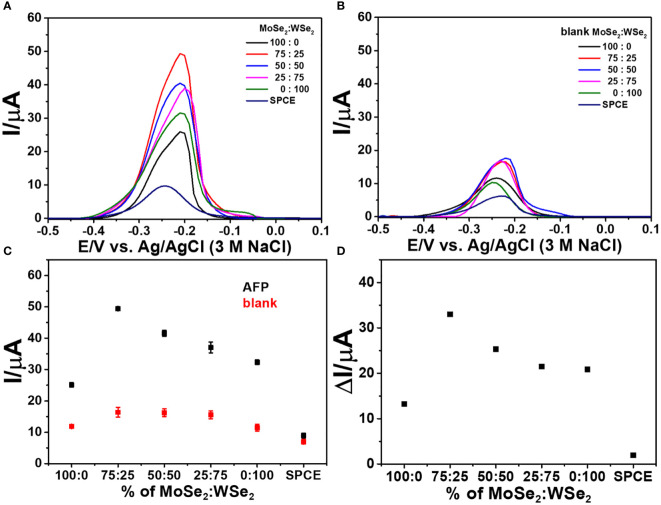
DPV responses of the sandwich-like immunosensors with different ratios of 2D MoSe_2_/2D WSe_2_ in contact with 0.010 M PBS (pH 7.4); **(A)** with and **(B)** without AFP after incubation with MB. **(C)** Corresponding current responses of panels (**A**, **B**, **D)** analytical responses or current differences of panel **(C)**.

Additionally, to choose good 2D materials for device fabrication, the analytical response of the developed immunosensor after capturing AFP (50 ng ml^-1^) is employed. As shown in **Figure 4**, the DPV peak current responses of immunosensors based on 2D MoSe_2_-, 2D WSe_2_-, and 2D MoSe_2_/2D WSe_2_ (75:25, 50:50, and 25:75% w/w)-modified SPCEs and bare SPCE were studied. The immunosensor’s construction parameters are anti-AFP, AFP, and MB concentrations of 50 µg ml^-1^, 50 ng ml^-1^, and 10 mM, respectively, and anti-AFP, AFP, aptamer (10 µM), and MB incubation times of 40, 40, 40, and 30 min, respectively. The DPVs of all biosensors were obtained in contact with 0.010 M PBS (pH 7.4). When the responses of the immunosensors made from the bare SPCE and single material (2D MoSe_2_ and 2D WSe_2_)- and 2D MoSe_2_/2D WSe_2_ (75:25, 50:50, and 25:75% w/w) composite-modified SPCEs are compared (**Figure 4**), the result shows that modification with the 2D MoSe_2_/2D WSe_2_ (75:25% w/w) composite gives the greatest DPV peak current after MB infiltration into the aptamer chain. Without AFP, the DPV peak currents of all immunosensors under the same preparation order are exhibited in **Figure 4**. All sensors reveal the significantly lower peak currents of MB compared to the sensors containing AFP (ca. 3.3 times for the current increment). The low response intensities of the aptamer-free immunosensors were due to no AFP increase using bare SPCE, single material (2D WSe_2_ and 2D MoSe_2_)-modified SPCEs, and 2D MoSe_2_/2D WSe_2_ (75:25, 50:50, and 25:75% w/w) composite-modified SPCEs. Due to the immunosensor without AFP capture, the aptamer cannot bind to the anti-AFP, and according to MB responses, the result implies an insignificant nonspecific adsorption of aptamer on the electrode surface. Consequently, **Figure 4** shows the comparison of current responses from immunosensors based on different materials after detection of AFP in 50 ng ml^-1^ AFP and blank solutions. Observable responses are lower for all blank measurements (no AFP), which agree with those in **Figures 3A** and **B**. It is found that the sensor using 2D MoSe_2_/2D WSe_2_ composite-modified SPCE (75:25% w/w) shows the highest peak currents for both cases. The corresponding current differences of the immunosensors with and without AFP are found as a function of the composition of electrode modifiers as shown in **Figure 4**. These differences are caused by different kinds of modified electrodes at the same AFP and aptamer concentrations. They are expected to be proportional to the AFP concentration when used as sensing signals. The highest current difference is obtained for the immunosensor based on 2D MoSe_2_/2D WSe_2_ (75:25% w/w). This result has good agreement with electrode property and reactivity (see CV and EIS results). Therefore, the 2D MoSe_2_/2D WSe_2_ (75:25% w/w) composite-modified SPCE is chosen as the electrode platform for the immunosensor fabrication to detect AFP throughout the device development.

**Figure 5:**
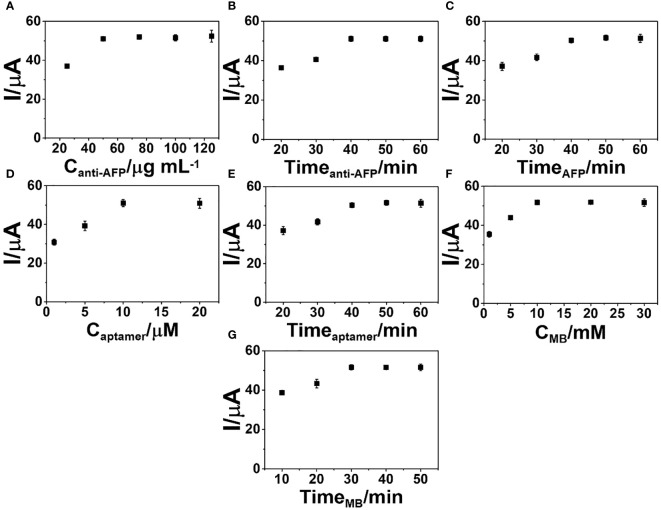
Optimization of the **(A)** anti-AFP concentration, **(B)** anti-AFP immobilization time, **(C)** incubation time for complete immunoreaction, **(D)** aptamer concentration, **(E)** incubation time of aptamer binding, **(F)** MB concentration for the signal generation, and **(G)** incubation time of MB intercalation into the sensing surface for the construction of the developed sandwich-like immunosensor.

To obtain the best sensitivity for the detection of the AFP protein, procedure parameters of the immunosensor fabrication were optimized such as concentrations of anti-AFP, aptamer, and MB, reaction periods of anti-AFP (immobilization on the electrode surface), aptamer (binding to captured AFP), and MB (interaction with the DNA aptamer structure), and immunoreaction time. **Figure 5** invesigates the behaviors of the graphs for the optimization. All show an increase in current response and reach a plateau against each parameter. **Figure 5** exhibits the electrochemical responses of the selected electrodes modified with various anti-AFP concentrations (25, 50, 75, 100, and 125 µg ml^-1^) for the detection of 50.0 ng ml^-1^ AFP using constant concentrations of aptamer (10 µM) and MB (10 mM) and constant times of 40, 40, 40, and 30 min for the incubation with anti-AFP, AFP, aptamer, and MB, respectively.

After conducting all of the immunoassay processes, the DPV peak current starts a constant at the concentration of 50 µg ml^-1^ anti-AFP, indicating that anti-AFP is saturated on the 2D MoSe_2_/2D WSe_2_-modified SPCE. Thus, the anti-AFP concentration of 50 µg ml^-1^ is employed in the next experimental study. Furthermore, the impact of anti-AFP reaction time for the anti-AFP immobilization onto the modified SPCE on the immunosensor performance is investigated over time ranges of 20–60 min, as illustrated in **Figure 5**. The optimization of the immobilization time for anti-AFP employs constant concentrations of anti-AFP (50 µg ml^-1^), AFP (50 ng ml^-1^), aptamer (10 µM), and MB (10 mM) and constant incubation times of 40, 40, and 30 min for binding AFP, aptamer, and MB onto the electrode, respectively. The immunosensor’s peak current increases, and no current change is observed after incubation of 40 min, suggesting complete reaction and saturation of the electrode surface with antibodies. Thus, an incubation time of 40 min for anti-AFP immobilization is selected for the next optimization. **Figure 5** illustrates the effect of AFP incubation time on the DPV peak current of the immunosensor in detecting 50 ng ml^-1^ AFP. After achieving the anti-AFP-saturated SPCE, the immunoassay is performed by covering with the AFP solution at the same aptamer and MB concentrations and the same incubation times for the aptamer and MB loadings. Incubation periods from 20 to 60 min for the complete immunoreaction are studied. It is found that when the incubation time is increased, the peak current intensity is steady from 40 min to higher values. As a result, a 40-min incubation period is used as an optimal interaction time for further study.

To find the best signal amplification, the response of the immunosensor after MB uptake is evaluated. The concentration of the aptamer as a parameter for signal generation is studied. Various aptamer solutions (1, 5, 10, and 20 µM) were employed in this study. Covering different amounts of aptamer, **Figure 5** displays the current responses of the immunosensors constructed under the condition above for detection of 50 ng ml^-1^ AFP. The greatest peak current is observed when using the aptamer concentration from 10 to 20 µM, suggesting a sufficient amount of aptamer, and the aptamer is fully immobilized on the electrode surface. After this point, there is an aptamer excess, which would cause a costly device. For the immunoassay, a 10-µM solution is chosen. The study of time for aptamer binding on the biosensors is required to obtain a stable response due to a complete aptamer/AFP reaction. The incubation time of the aptamer solution on the immunosensor was then studied over many periods of time (20–60 min). Under an optimized fabrication parameter for detection of 50 ng ml^-1^ AFP, the peak current reaches a maximum point at the incubation time of 40 min and then it is constant, as shown in **Figure 5**, resulting from the complete binding between the captured AFP and aptamer. A 40-min incubation time is achieved as the minimal time in the immobilization of the aptamer for intercalation of the signaling MB molecules.

As presented in **Figure 5**, the MB concentration (1–30 mM) for the production of the best electrochemical signal of MB collected on the aptamer-bound immunosensor was also determined. With the constant incubation time of MB solution at 30 min, the developed sandwich-like immunosensor fabricated from the same condition above shows that MB concentrations from 10 mM offer the best response for detection of 50 ng ml^-1^ AFP. In this study, the MB concentration of 10 mM is chosen for signal generation. Furthermore, to obtain the best signal generation, the incubation period (10–50 min) for the redox probe (MB), completely attached to the captured aptamer chain on the target AFP, is studied. As displayed in **Figure 5**, under the same fabrication process, the incubation time of 30 min is the suitable time to fully complete the MB intercalation for the proposed sensor in the assay of 50 ng ml^-1^ AFP. Again, to achieve a great sensor, the optimized fabrication parameters are anti-AFP concentration of 50 µg ml^-1^, aptamer concentration of 10 µM, and MB concentration of 10 mM, as well as incubation times of 40, 40, and 30 min for the immobilization of anti-AFP, aptamer, and MB, respectively, and a 40-min AFP incubation time.

### Performance of the immunosensor based on the 2D MoSe_2_/2D WSe_2_ composite

**Figure 6:**
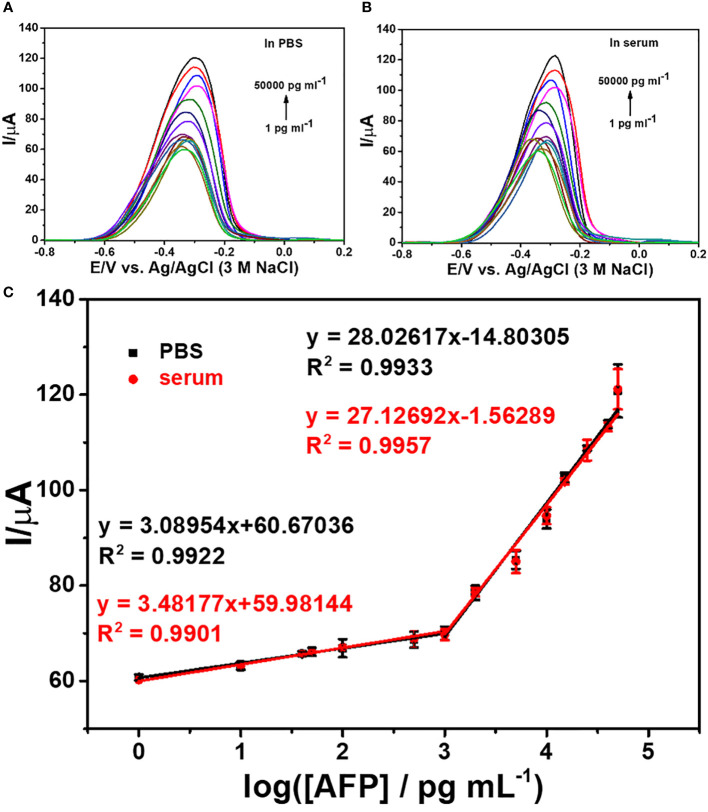
DPV responses of the immunosensors after incubation with AFP in PBS **(A)** and 50-fold diluted human serum **(B)** at different concentrations and **(C)** logarithmic calibration curves of the two matrix systems.

DPV is used to measure the electrochemical responses of the MB/aptamer/AFP/BSA/anti-AFP/2D MoSe_2_/2D WSe_2_-modified SPCE after incubation with different AFP concentrations (1–50,000 pg ml^-1^) in 0.0010 M PBS (pH 7.4), as shown in **Figure 6**. An increase in the peak current of MB oxidation at a potential of -0.35 V is found with increasing AFP concentrations. The current has a linear relationship to the logarithmic AFP concentration. Furthermore, **Figure 6** shows the corresponding calibration curve composed of two different concentration ranges (1–50 and 50–50,000 pg ml^-1^). The linear regressions are *I* (μA) = 3.09log*C*_AFP_ + 60.67 (*R* ^2^ = 0.99) and *I* (μA) = 28.03log*C*_AFP_ − 14.80 (*R* ^2^ = 0.99), respectively. An LOD of 0.78 pg ml^-1^ for the detection of AFP in PBS is obtained. In a 50-fold diluted human serum (Phetsang et al., 2021; Yaiwong et al., 2021), the electrochemical immunosensor is evaluated using DPVs for assays of various spiked AFP concentrations (1-50,000 pg ml^-1^) as shown in **Figure 6**. The response of MB/aptamer/AFP/BSA/anti-AFP/2D MoSe_2_/2D WSe_2_-modified SPCE reveals a similar behavior as that in **Figure 6**. Linear equations for the calibration curve in the diluted serum (**Figure 6**) are *I* (μA) = 3.48log*C*_AFP_ + 59.98 (*R* ^2^ = 0.99) and *I* (μA) = 27.13log*C*_AFP_ − 1.56 (*R* ^2^ = 0.996) for concentration ranges of 1–50 and 50–50,000 pg ml^-1^, respectively, with an LOD of 0.85 pg ml^-1^. Both calibration curves can be fully superimposed, indicating no significant difference between the detections in PBS and diluted serum. This also implies that the proposed biosensor has high selectivity in the human serum matrix since it contains real interferences (Pothipor et al., 2019). From this result, the immunosensor can be used in the real-world clinical AFP assay. The comparison of several electrochemical immunosensors for the detection of AFP is shown in **Table 1**. It is noted that the developed immunosensor has an acceptable low LOD and wide dynamic range that is adequate for the detection of AFP in diagnosing liver cancer. As compared to the first sensor, although its LOD is extremely lower, our immunosensor presents less complexity for signal generation and a wider dynamic range. Furthermore, Sensor 3 demonstrates the tag consisting of polymer that the synthetic process is time-consuming and has a higher LOD. LODs for Sensors 2 and 5 are higher than that of our sensor, they also demonstrate complicated electrochemical platforms, and Sensor 5 uses a complex tag. In addition, Sensor 4 employs a simple tag and electrode. For the signal amplification, Sensors 2 and 4 require the enzymes’ substrates while Sensor 5 needs the catalyst’s substrate. The tags of Sensors 2 and 4 would be enviromentally sensitive because of the nature of the enzymes. Therefore, the drawbacks of the five reported sensors limit their applicability for the AFP assay.

**Table 1:** Comparison of detection performance of the proposed biosensor with various AFP immunosensors.

Platform	Tag	Method	Linear range (ng ml^−1^)	LOD (ng ml^−1^)	Ref.
Ab_2_/AuNPs/GCE	Pd/APTES-M-CeO_2_-GO-Ab_2_	Amp	0.0001-50	3.3×10^-6^	(Wei et al., 2016)
Ab_1_/GO-MB-AuNPs/GCE	AuC-HRP-Ab_2_	DPV	0. 005-20	1.5×10^-3^	(Shen et al., 2020)
Ab_1_/AuNPs-GO/GCE	P(VT-co-HEMA)-g-GO/Ab_2_	SWV	0.025 - 50	1.8×10^-2^	(Zhao et al., 2020)
Ab_1_/3D AuE	HRP/Ab_2_	Amp	0.005-50	3.0×10^-3^	(Zhong et al., 2015)
Ab_1_/rGO-TEPA-Thi-AuNPs/SPCE	CMK-3@AuPtNPs-Ab_2_	Amp	0.005-100	2.2×10^-3^	(Xiao et al., 2021)
Ab_1_/2D MoSe_2_/2D WSe_2_/SPCE	MB/aptamer	DPV	0.001-0.050 0.050-50	8.5×10^-4^	This work

GCE, glassy carbon electrode; AuNP, gold nanoparticle; AFP, alpha-fetoprotein; Ab_1_ and Ab_2_, anti-AFP antibody; BSA, bovine serum albumin; GO, graphene oxide; M-CeO_2_, cerium oxide mesoporous nanoparticles; APTES, 3-aminopropyltriethoxysilane; Pd, palladium; MB, methylene blue; HRP, horseradish peroxidase; AuC, gold cube; P(VT-co-HEMA), poly(vinyltetrazole-co-hydroxyethyl methacrylate); 3D AuE, three-dimensional gold electrode; rGO, reduced graphene oxide; TEPA, tetraethylene pentamine; Thi, thionine; CMK-3, mesoporous carbon; PtNP, platinum nanoparticle; SPCE, screen-printed carbon electrode; Amp, amperometry; DPV, differential pulse voltammetry; SWV, square-wave voltammetry.

### Selectivity, reproducibility, and stability of the immunosensor

**Figure 7:**
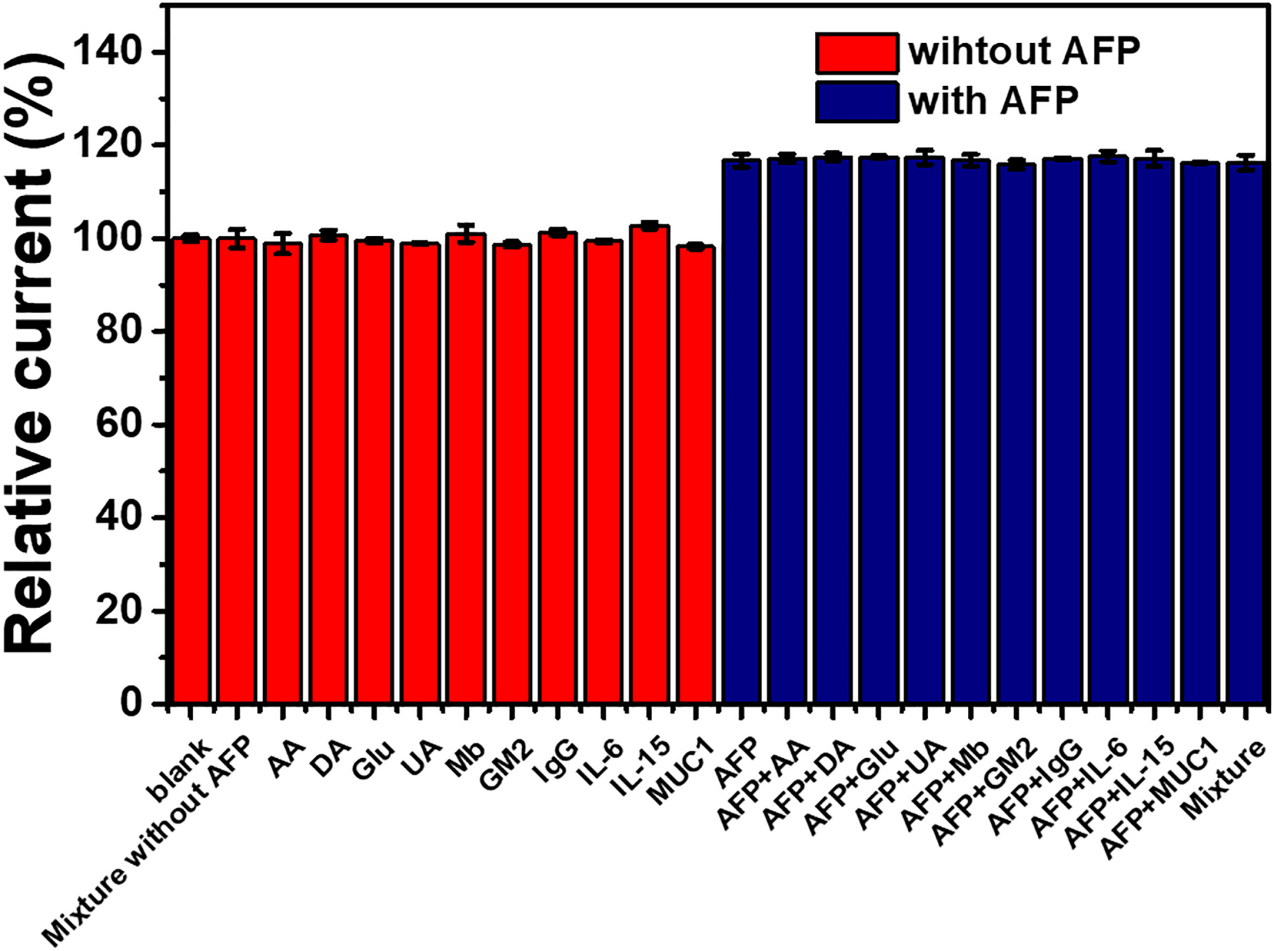
Interference study of the immunosensor incubated with different solutions: blank (PBS), individual and mixed interference solutions (100 ng ml^-1^), and 1 ng ml^-1^ AFP solutions without and with the presence of individual and mixed interferences (100 ng ml^-1^).

**Figure 8:**
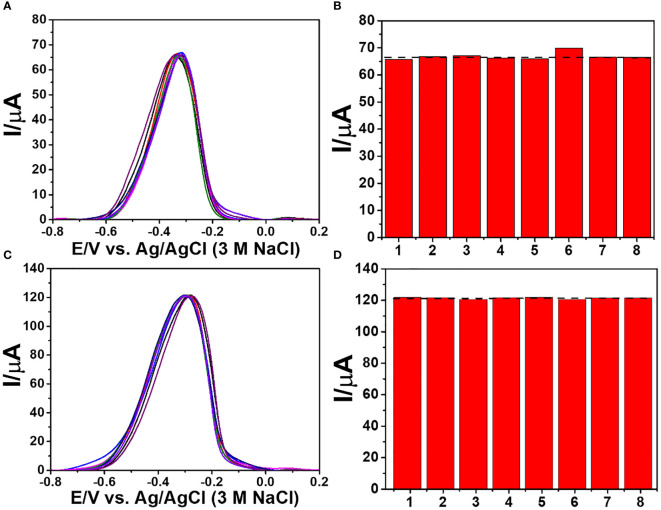
Reproducibility study of the immunosensor for the detection of 0.10 and 50.0 ng ml^-1^ AFP; **(A, C)** DPV sensorgrams and **(B, D)** corresponding current responses.

**Figure 9:**
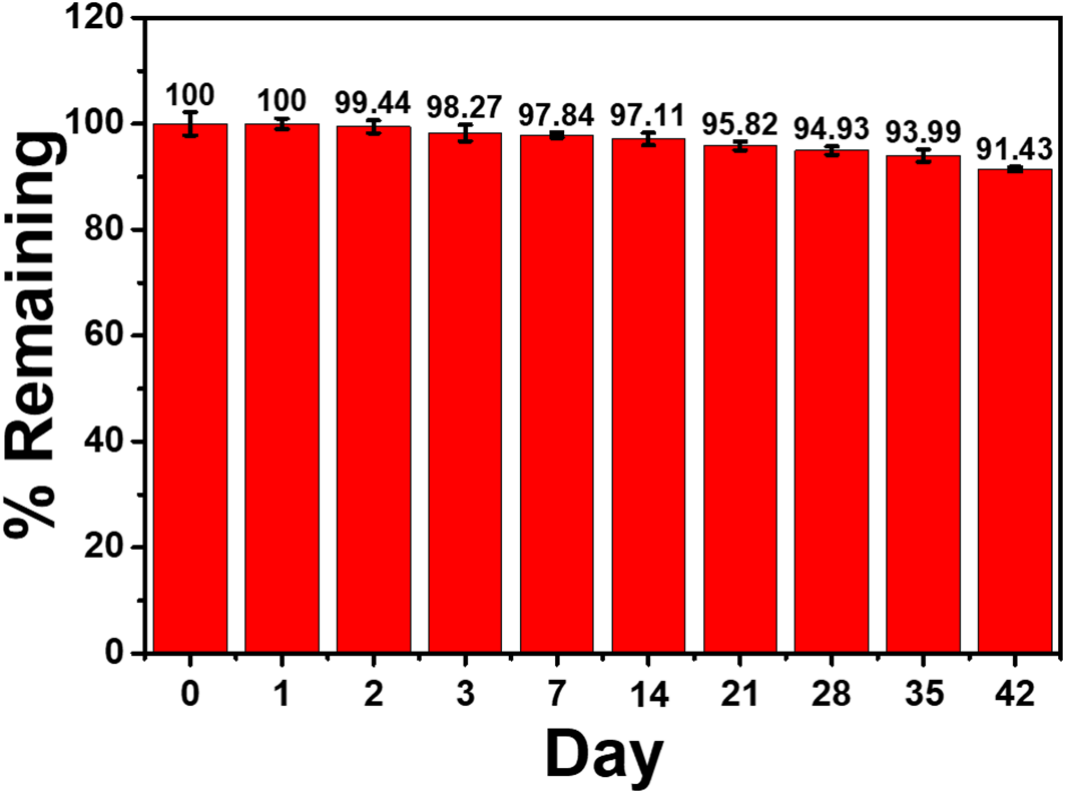
Stability of the electrochemical immunosensor in the detection of AFP.

To test the immunosensor’s specificity, possible interferences including redox and non-redox molecules such as AA, DA, Glu, UA, Mb, GM2 activator protein (GM2), IgG, IL-6, IL-15, and MUC1 were used. There are two groups of solutions; the first group involves a blank solution and AFP-free solutions with the presence of individual AA, DA, Glu, UA, Mb, GM2, IgG, IL-6, IL-15, MUC1, and their mixture (100 ng ml^-1^), while the second group invloves an interference-free AFP solution (1.0 ng ml^-1^) and 1.0 ng ml^-1^ AFP solutions containing the individual and mixed interferences at the 100-fold concentration. After the immunosensor was incubated with the solutions, the current responses are obtained in **Figure 7**. The result shows that the solutions containing 1.00 ng ml^-1^ AFP give higher responses than those of the solutions with no AFP; however, the currents are not significantly different among the presence of the individual and mixture interferences, suggesting high device selectivity. Moreover, the AFP-free solutions of individual interferences and their mixture provide insignificantly different current responses as compared to that of blank solution, thus implying no occurence of nonspecific adsorption. Therefore, at the extremely higher concentration, the potential interferences and interference mixture could not affect the analytical signal, resulting in high selectivity and good applicability of the proposed immunosensor. This agrees well with the detection study above (superimposition of calibration curves for the AFP detection in PBS and diluted human serum in **Figure 6**) (Pothipor et al., 2019). Reproducibility is also a critical parameter for the scale-up production of immunosensors and is examined in order to ensure the reliability of this new immunosensor. The AFP assays in 0.10 and 50.0 ng ml^-1^ AFP solutions were tested, and each assay was carried out using eight individual similarly prepared immunosensors. **Figures 8A**–**C** illustrate their sensorgrams, and **Figures 8B** and **D** represent the corresponding peak currents (ca. 66.35 and 121.40 µA), respectively. It is noted that no significant difference is observed in the current responses. The relative standard deviations (RSDs) obtained for the eight individual constructed immunosensors in detecting 0.10 and 50.0 ng ml^-1^ AFP are 0.70% and 0.43%, respectively. As a result, the immunosensor’s precision and reproducibility are acceptable. The immunosensor’s stability was tested by measuring the current response in the detection of 10 ng ml^-1^ AFP after storage from 1 to 42 days. When not in use, the immunosensor was kept at 4°C in a moisture chamber. Six individual immunosensors at each storage period were tested, and the result is shown in **Figure 9**. After the 3-week storage, the immunosensor’s current response is changed by ca. 4.2%. After 6 weeks, the current response remains at 91.4% of its original current, indicating that the immunosensor is sufficiently stable.

### Application of the immunosensor

To assess the analytical reliability and application potential of the proposed immunosensor, analyzing the AFP level in human serum to find the recovery amount is studied. An AFP standard solution was added into and diluted with a 50-fold diluted human serum solution (Phetsang et al., 2021; Yaiwong et al., 2021) to obtain four different final concentrations (1, 5, 10, and 25 ng ml^-1^). The detection of AFP in such solutions is performed using the immunosensor. As listed in **Table 2**, percentage recovery and %RSD values range from 95.39% to 102.1% and 0.66% to 1.93%, respectively. The results suggest acceptable applicability of the immunosensor in clinical analysis.

**Table 2:** Recovery study for the determination of AFP in human serum by the present immunosensor.

Sample	Standard of AFP (ng ml^-1^)	Found (ng ml^-1^)	Recovery (%)	RSD (%)
1	1	1.02 ± 0.29	102.1	0.82
2	5	4.77 ± 0.98	95.39	1.93
3	10	9.98 ± 0.71	99.75	1.48
4	25	24.62 ± 0.71	98.47	0.66

AFP, alpha-fetoprotein; RSD, relative standard deviation.

## Conclusions

This research work creates a new portable sandwich-like electrochemical immunosensor based on a 2D nanomaterial composite for the quantitative detection of AFP using a signaling MB/aptamer complex. A 2D MoSe_2_/2D WSe_2_-modified SPCE as an electrochemical sensing platform gives good device performance in detection. The 2D MoSe_2_/2D WSe_2_ provides an improvement in electrode reactivity and kinetics. The increment of current response correlates with the amount of target AFP because of specific bindings of aptamer to the captured AFP and MB. The suggested immunosensor has two wide linear ranges (1–50 and 50–50,000 pg ml^-1^) with a low LOD of 0.85 pg ml^-1^, high reproducibility, exceptional selectivity, and acceptable stability. The strategy for fabrication of the developed biosensor also offers good device sensitivity, shorter analytical time, and cost-effectiveness for clinical analysis. The immunosensor from this study is an alternative tool to detect the AFP biomarker for clinical liver cancer diagnosis and monitoring, and it can be further developed for the electrochemical assays of other tumor indicators.

## Data availability statement

The original contributions presented in the study are included in the article/**Supplementary Material**. Further inquiries can be directed to the corresponding author.

## Author contributions

SC: writing—original draft, investigation and data curation; JJ: writing—review and editing; KO: conceptualization, methodology, formal analysis, resources, validation, writing—original draft, writing—review and editing, supervision, project administration, and funding acquisition. All authors contributed to the article and approved the submitted version.

## Funding

This research projectwas supported by Fundamental Fund 2022, Chiang MaiUniversity (CMU).

## Acknowledgments

The authors gratefully acknowledge Research Center on Chemistry for Development of Health Promoting Products from Northern Resources, Center of Excellence for Innovation in Chemistry (PERCH-CIC), and Faculty of Science, CMU.

## Conflict of interest

The authors declare that the research was conducted in the absence of any commercial or financial relationships that could be construed as a potential conflict of interest.

## Publisher’s note

All claims expressed in this article are solely those of the authors and do not necessarily represent those of their affiliated organizations, or those of the publisher, the editors and the reviewers. Any product that may be evaluated in this article, or claim that may be made by its manufacturer, is not guaranteed or endorsed by the publisher.
