# Supplementary material for: A sandwich-like configuration with a signal amplification strategy using a methylene blue/aptamer complex on a heterojunction 2D MoSe2/2D WSe2 electrode: Toward a portable and sensitive electrochemical alpha-fetoprotein immunoassay
Source: Front Cell Infect Microbiol. 2022 Oct 27;12:916357. doi: 10.3389/fcimb.2022.916357 (PMC9646986; doi:10.3389/fcimb.2022.916357)
Supplement: Supplementary file 1 [file DataSheet_1.docx]

*Supporting Information*

A Sandwich-like Configuration with a Signal Amplification Strategy Using a Methylene Blue/Aptamer Complex on a Heterojunction 2D MoSe_2_/2D WSe_2_ Electrode: Toward a Portable and Sensitive Electrochemical Alpha-fetoprotein Immunoassay

Supakeit Chanarsa^1,2^, Jaroon Jakmunee^1,2,3^, Kontad Ounnunkad^1,2,3^*

^1^Department of Chemistry, Faculty of Science, Chiang Mai University, Chiang Mai 50200, Thailand

^2^Center of Excellence for Innovation in Chemistry, Faculty of Science, Chiang Mai University, Chiang Mai 50200, Thailand

^3^Research Center on Chemistry for Development of Health Promoting Products from Northern Resources, Chiang Mai University, Chiang Mai 50200, Thailand

*** Correspondence:**Corresponding Author
kontad.ounnunkad@cmu.ac.th

suriyacmu@yahoo.com


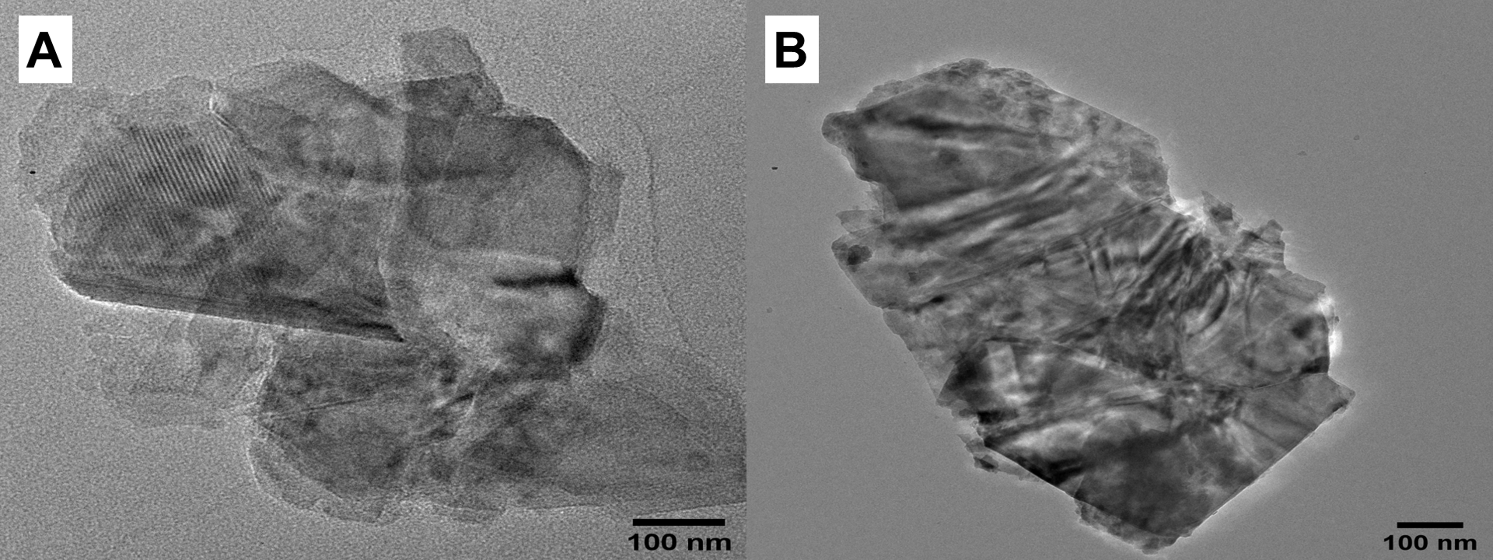


**Figure S1** TEM images of MoSe_2_ (**A**) and WSe_2_ (**B**)
